# A Rigorous and Integrated On-Water Monitoring System for Performance and Technique Improvement in Rowing

**DOI:** 10.3390/s23136150

**Published:** 2023-07-04

**Authors:** Thanassis Mpimis, Vassilis Gikas, Vassilios Gourgoulis

**Affiliations:** 1 School of Rural, Surveying and Geoinformatics Engineering, National Technical University of Athens (NTUA), 15780 Zografos, Greece; vgikas@central.ntua.gr; 2 School of Physical Education and Sport Science, Democritus University of Thrace (DUTh), 69100 Komotini, Greece; vgoyrgoy@phyed.duth.gr

**Keywords:** biomechanics, kinetics, kinematics, stroke cycle, GNSS/INS

## Abstract

This paper presents a prototype, on-water rowing monitoring system and its testing results for a single scull boat. The proposed system aims at recording critical kinetic (athlete biomechanics and oar/seat movements) and kinematic (boat position, velocity, acceleration, and attitude) parameters for sport performance evaluation and rowing technique improvement. The data acquisition unit is organized in two parts: the first part aims at logging boat kinematics based on GNSS/INS filtering, while the second one facilitates kinetics data recording using a series of analog sensors (potentiometers, strain gauges) installed on the athlete’s body and the boat seat and oars. Both parts are connected to a central unit featuring analog voltage digitizers and a micro-PC for device handling and data storing. In order to test the performance of the system a series of field trials were undertaken featuring different observation scenarios as well as intentionally induced errors in the rowing technique. Analysis revealed the high performance of the system in terms of sensor completeness and setup procedures as well as operational efficiency. Moreover, system performance evaluation exercised through studying raw data recordings and resultant parameters at stroke cycle and average (standardized) stroke cycle level confirmed the fruitfulness of the proposed approach and system and its potential for implementation on a broad scale. Finally, the data acquired from the proposed system were used to compute the adopted input parameters and performance indicators to characterize the system in terms of functionality and operational efficiency.

## 1. Introduction

Regardless of the distinguishing characteristics of an individual sport, the ranking between the finisher and the successor is usually realized by very small differences in performance. In competitive rowing, the finish time of a typical 2000 m race is governed by many factors during the course. These influencing factors are broadly known as rowing kinetics [1,2]. They refer to biomechanical parameters concerned with the movement of the athlete’s body and the reaction of the boat equipment (oars, seat) to such input. As a result of the cumulative action applied by the athlete and the environmental effects, the boat moves forward. Its running state during the course is described by the instantaneous boat kinematics, namely, its position, velocity, acceleration, and attitude [3]. The simultaneous recording and analysis of the rowing kinetics and kinematics convey critical information for studying, characterizing, and improving the athlete’s technique.

In order to study in a holistic manner performance assessment in competitive rowing, studies from other scientific fields have focused on different aspects (e.g., analyzing the brain’s ability to maintain motor control and the body’s metabolism) [4,5]. These factors are crucial to the final performance in competitive rowing; however, they are beyond the scope of this study.

Recently, the rapid advancements in multi-sensor technologies and data processing techniques opened widely the road for the development of specialized monitoring systems to fulfill the requirements for numerous applications [6,7,8]. In this regard, when setting up a sensor platform to track a process, the selection of specific sensor types, their technical characteristics, and the operational/communication interface depends on a number of criteria dictated directly by application needs [9]. Similarly, the type of data analysis techniques (e.g., diagnostic, statistical, AI-based) to be adopted should match the nature, quality, and quantity of the recorded data to optimally describe the problem characteristics [10].

Despite the highly repetitive character and the fine (or even synchronized, in the case of a crew) movements required in competitive rowing, the relationship and dependence between kinetics and kinematics is a rather complex process. Therefore, in order to fully describe and understand the rowing problem and to extract the athlete’s technique (known also as the “athlete signature”), advanced sensor systems and sophisticated analysis techniques are deemed necessary. Nevertheless, despite the fact that numerous studies and various systems are available on the market today (see Section 2), the need for a fully comprehensive approach to the rowing problem is still evident.

This paper presents a conceptual approach and describes a rigorous and integrated monitoring system that consists of a sensors platform to capture the boat kinematics and athlete’s kinetics. The emphasis is placed on the design, the setup, and the testing of the integrated system in terms of correctness and operational efficiency. A computational approach for extracting and characterizing the athlete’s technique for a single scull is also introduced. The remainder of the paper is organized as follows. Section 2 provides the motivation and state of the art of this work. Section 3 offers a detailed description of the design and implementation of the system, while Section 4 discusses the results of the testing of the systems using a suitable set of field data. Finally, Section 5 provides conclusions and an outlook for performance modeling and technique extraction in rowing.

## 2. Background and Motivation

The interest in systematic monitoring (recording and analyzing) of the athlete’s performance in rowing dates back to the early twentieth century. Many researchers have referred to the parameters that determine the rowing process, including those related to biomechanics [10] and the physiology of the rower, as well as those related to psychology and sports medicine [11]. Nevertheless, most studies and performance monitoring systems in rowing are confined to the recording of basic biomechanical parameters [1,12]. Today, there exists a variety of contemporary, on-water apparatuses for rowing performance monitoring featuring different biomechanical sensors as they are becoming increasingly popular. Such data sources can be used to measure the forces acting on the rower and the boat, providing valuable information to the coach and the rowing team to assist them in optimizing the training regimens and improving the technique while preventing injuries and thus ultimately leading to better performance levels.

These systems, however, usually provide specific measurement data by using rather simplistic statistical analysis tools for supporting the coach and the athletes during the training process. Moreover, there exist more complex systems [2,13] based on field rowing data, experimental data obtained from ergometers [14,15,16,17,18,19], or even simulation data representing the rowing process [20,21,22,23,24,25,26,27,28,29,30].

Regardless of the specific goals of each study, most available systems focus on the parameters concerned with the performance of a certain subsystem of the overall problem, such as the boat, the rowing equipment, or the athlete [29,31,32,33,34,35,36,37,38,39,40,41]. Today, only a limited number of studies present measured results from multi-sensor/multi-purpose monitoring systems [10,42,43,44]. However, in most cases the recorded data are treated independently, while conclusions are drawn on the basis of comparing raw data rather using a fully integrated scheme.

As an example, in [40] a wireless sensor network (WSN) was introduced. This system captures the kinematic and kinetic characteristics of the boat and the oars, respectively; however, it fails to describe the kinematic characteristics of other elements of the boat equipment, as well as characteristic joint movements of the athlete. A more comprehensive recording system was proposed in [9,10], offering the possibility of easy installation on the rowing boat. However, this system does not record the joint angles specific to standard movements of the athlete. Furthermore, it does not record the position and kinematics of the vessel, except for the acceleration, through which the boat’s sailing speed is calculated.

A network of three synchronized inertial sensor units conveniently placed on board was presented in [45]. The result of the recording is the calculation of the paddle length as well as the rowing rate. Some critical parameters in rowing training are computed; nevertheless, the rowing problem is not fully addressed. Another study applied to the sport of kayaking was presented in [46], the main characteristics of which could be easily adapted to the rowing case. This study employed strain gauges, inertial sensors (accelerometers, gyroscopes, and magnetometers), a GNSS receiver, and a microcontroller to synchronize the observations. However, angles at characteristic joints in the athlete’s body were not recorded. The authors in [47] focused on capturing the main phases of the rowing cycle using a smart mobile phone. The proposed methodology improves the accuracy of low-cost sensors embedded in modern smart phones (GPS satellite receiver and accelerometers) to compute the vessel’s speed, the total distance traveled, as well as the sailing distance per paddle cycle.

In competitive rowing, the finish time of a typical 2000 m race depends on a number of continuously interacting factors during the course. Ignoring environmental factors, the influencing factors of prime interest can be attributed to the rower and boat equipment and can be grouped into two main categories as follows:

- The boat subsystem, being responsible for the parameters concerned with the rowing boat equipment and the technique the athlete applies,
- The athlete subsystem, which refers to the biomechanical parameters affecting the athlete’s body movements.

In order to capture and improve the rowing technique, it is important to map the relationship between the athlete input (action) and boat kinematics (reaction). More specifically, the critical parameters identified as determinants for the evaluation of the rowing technique are classified in Table 1.

In this regard, this paper develops along two main axes:

- The conceptualization, testing, and adoption of an individual set of parameters that can reliably describe the rowing technique and standardize the classification of rowing performance,
- The construction and testing of an on-water, portable monitoring system for a single scull aiming at recording all necessary variables to provide sufficient data for computing the parameters of the previous step.

## 3. Conceptualization and Implementation of Proposed On-Water Rowing System

**Figure 1:**
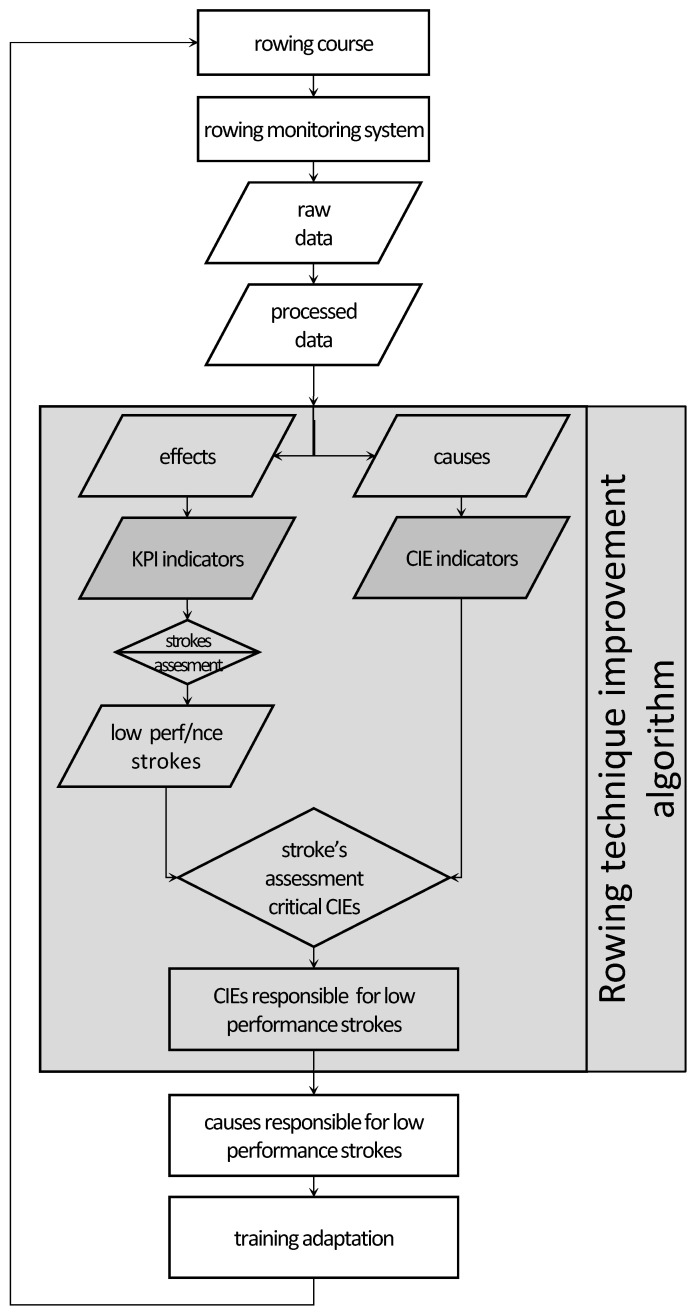
Algorithmic layout for technique monitoring and improvement in rowing.

The ultimate goal behind this approach is to improve performance indicators and thus to identify the causes that produce low-performing rowing cycles. The rowing stroke cycle is an iterative process for which critical parameters take repeated values within a range and around an equilibrium value, which changes per stroke cycle. To facilitate our goal, a performance improvement algorithm is proposed that uses summary indicators of the technique and performance in each rowing cycle (Figure 1).

These indicators are defined in such a way that they summarize the rowing stroke cycle using measured critical parameters. More specifically, the measured quantities are grouped in two categories: one concerned with the causes affecting the boat (applied technique), and a second one concerned with the response of the rowing boat to the causes (performance). The critical indicators used to describe the first category are called Critical Input Elements (CIEs), while the corresponding performance indicators are called Key Performance Indicators (KPIs).

As part of the performance improvement algorithm, the stroke cycles featuring low performance were selected by applying classification to KPI indicators. For the selected stroke cycles using CIEs, the measured parameters that were most responsible for the low performance can be estimated. Thus, the athlete’s training procedure is personalized according to its unique imprint as recorded by the proposed rowing monitoring system and errors in technique as detected by the performance improvement algorithm.

### 3.1. Parameters for Summarizing Performance and Technique

Rowing is a periodic movement since the rowing stroke cycle shapes up in four separate phases: catch, drive, finish, and recovery. The catch involves placing the oar blade in the water. Then, in order to move the boat, the rower drives the oars against the foot stretchers to pull the blade through the water. The finish is defined by the removal of the blade from the water. During recovery the rower moves slowly back up the slide toward the catch and feathers the oar blade so it is perpendicular to the surface of the water, ready for the next drive. Clearly, in order to achieve a good rowing performance, efficient but consistent stroke-to-stroke paddling is deemed necessary.

#### 3.1.1. Key Performance Indicators (KPIs)

In this section, the results related to the evaluation criteria of the athlete’s performance based on the KPIs for the rowing data collected are defined, described, and presented. KPIs describe in a systematic and standardized way the degree of performance of the athlete reduced to the scale of a rowing stroke cycle or for the entire course.

In essence, the KPIs reflect the efficiency of the rower’s technique, which summarizes the characteristics of the rower’s course and performance. The computation of parameters in this category relies primarily on the boat’s localization features, which are appropriately transformed to reflect motion characteristics along and across the vessel motion. Then, through statistical processing, two evaluation levels are obtained: (a) for each individual stroke cycle of a course, and (b) for the mean stroke of a route as a representative–a characteristic stroke cycle for all.

The KPIs herein are divided into two main categories as follows:

1. Rowing boat kinematics parameters

Extra distance (cm): refers to the additional length that the boat travels during a course owing to the deviation of its trajectory from the ideal straight course. The ideal course is defined by the starting point and the end of the course.

Linear deflection (cm): refers to the average transverse deflection of the boat (mean value and standard deviation) from the ideal course.

Angular deflection (arcmin): refers to the average angular deflection of the boat’s course direction (mean value and standard deviation) from the ideal course direction.

Additional time (ms): refers to the additional time required to cover the extra distance. It is obtained by dividing by the average speed of the boat per oar cycle.

2. Rowing technique parameters

Stroke length (m): estimation of the travel length for each stroke cycle in terms of rowing stroke rate.

Velocity fluctuation (m/s): refers to the difference between the mean and the minimum stroke cycle speed.

Track deflection (m): refers to the surface enclosed between the actual and the ideal trajectory of the boat. To perceive and visualize it, it is divided by the ideal course length.

#### 3.1.2. Critical Input Elements (CIEs)

The parameters that describe the rowing technique are called Critical Input Elements (CIEs). CIEs describe in a quantitative and explicit way the fundamental movements of the athlete and the boat equipment during a course. The information used to form the CIEs comes from the kinematic and kinetic data of the athlete’s body, the oars, and the hull of the boat.

Geometric quantities concerned with the equipment size and its location on the boat as well as somatometric figures are defined that summarize the athlete’s technique. A characteristic of these quantities in each stroke cycle is that they vary over a range of values (e.g., horizontal paddle angle of about [−60, 40] deg) and are not characterized by any fixed equilibrium value.

The CIEs adopted in this study are divided into two categories:

1. Parameters of regularity (symmetry and synchronization) during rowing technique:
  - Difference in the horizontal angles between the left and right oar in the transition phases during the “catch”, “drive”, and “finish” versus the time lag of their achievement (deg versus ms); indicates the degree of symmetry and synchronization of the horizontal movement of the oars during the stroke cycle. This results in three pairs of values.
  - Synchronization of the vertical angle between left and right oars during the application of maximum force into the stroke cycle (deg): indicates the degree of symmetry and synchronization of the sunk oars at the moment of the application of the maximum force in the stroke.
  - Symmetry of the horizontal and vertical angles for each oar at the moment of application of the maximum force during the stroke cycle (deg versus deg): indicates symmetry for the positions of the oars at the moment of application of the maximum force on the strokes.
  - Symmetry of the maximum application force to the stroke between the left and right oars during the “drive” phase (N): indicates the degree of symmetry and synchronization of the maximum force applied by the athlete through the oars to the gate.
2. Parameters of incorrect technique applied at the rowing cycle:
  - Incorrect sequence of movements during the “drive” phase of the stroke cycle (cm): the correct sequence of engagement of the three main muscle groups of the rower’s body in order to produce force during the drive phase is not maintained. The correct sequence dictates first the use of the quadriceps of the legs (legs), the back, and finally the biceps of the arms (arms). Thus, the correct sequence is legs–back–arms, and an error in this sequence will cause a regression of the athlete’s seat at the end of driving phase.

### 3.2. System Design

To achieve the goal of the proposed algorithm, it is necessary to calculate the KPI and CIE parameters as described in Figure 1. The data required for the calculation of these indices comes from an on-water rowing monitoring system that is adapted to the boat, its equipment, and also to the rower. Details on the design of this system are included in this chapter.

#### 3.2.1. System Requirements

In the context of this research, a prototype model of an on-water system for monitoring rowing performance was developed. In order to ensure the completeness (in terms of observation types and their distribution) and operational efficiency (accuracy, availability, robustness, etc.) of the proposed system, a minimum set of design requirements need to be met.

On one hand, robustness is a prerequisite for the reliable operation of the system in real and tough outdoor conditions. On the other hand, high-quality measurement assurance, data completeness, and synchronization are necessary for the reliable assessment of the athlete’s technique based on field data.

1. System prototype functionality/durability: The proposed system has to be as light as possible while offering a high degree of autonomy to ensure smooth operation, even in abrupt operational conditions.
2. Requirements for high-quality field measurements: High precision, high reliability of synchronized measurements, and high data availability are critical in order to monitor and successfully extract the athlete’s technique.

The specifications examined in order to achieve the most accurate digitization are: (a) Analog to Digital resolution (bits), (b) maximum digitization rate, and (c) simultaneous digitization between different digitization channels.

#### 3.2.2. Sensor Types and Technical Specifications

In order to successfully monitor a rowing event a number of different types of sensors are required to be placed across the entire platform (rower, boat, oars) in motion. There exist different categories of sensors depending on the installation environment, the desired accuracy, and the user interface. Clearly, the appropriate technical specifications should be defined, which will lead to the selection of the most suitable sensors for the current application. A key design specification of the proposed system assumes that boat kinematics are acquired independently from other parameters. In this regard, a separation between logging subsystems is also applied. The first logging system concerns the acquisition of the boat’s kinematics, while the second subsystem is dedicated to kinetics data associated with the athlete and boat equipment.

1. The boat’s kinematics acquisition subsystem

The reliable loggings of the boat’s kinematics require the combination of a satellite-positioning receiver, inertial sensors, and their processing via a real-time Kalman filter. The development of related systems is a solved problem in the era of kinematic localization and navigation and is not the aim of the current research. Thus, a commercial GPS/INS system was chosen to acquire the boat’s kinematic characteristics. The basic conditions imposed for the selection of such system are: (a) that it be lightweight, portable, and water-resistant; (b) that it has synchronization ability with the measurements of the other parts of the overall recording system; and (c) that it be low cost.

2. The athlete’s and boat’s equipment kinetics acquisition subsystem

The specifications associated with the design and performance of this subsystem are particularly demanding compared to those concerned with the boat’s kinematics. More specifically, technical limitations must be considered in relation to the way the sensors can be adapted and installed on the boat equipment as well as on the rower’s body. Considering that the raw data originate from variant sensors, their sampling rates and time synchronization are vital. Obviously, simultaneous data collection by all sensors cannot be a prerequisite—such problems were dealt with in the pre-processing software.

For the second subsystem that logs kinetics data, all contributing sensors are analog. Their specifications relate to the individual parameter observed as follows:

- Applied force on the oar rings: potentiometer bearing maximum dimensions of 7 × 5 × 10 mm (L × W × H), so that it can be integrated into the oar lock by the gate point of the boat. It features a measurement range up to 1 kN (about 100 kg), is waterproof, and accounts for an accuracy better than 10 N (approx. 1 kg).
- Applied force at the soles of the rower’s feet: piezoelectric bearing area dimensions in the range of a typical foot (~25 × 15 cm) and thickness ≤1 mm, easily adjusted to fit on the shoes fixed on the boat, be flexible enough to adapt to the shape of the sole, be waterproof, have a measuring range of up to 50 kg per sole, and measure force with an accuracy better than 1 kg.
- Linear position of the athlete’s seat: lightweight potentiometer, easily fitted under the rails of the seat, water-resistant, featuring a minimum measuring range of 0.70 m and a minimum accuracy of 1 mm.
- Oar angle measurement sensor: potentiometer bearing the maximum physical dimensions of 25 × 25 × 25 mm (L × W × H) to allow integration in the oar lock by the boat gate, waterproof, featuring a minimum angle measurement range of 120 deg and an accuracy of at least 1.0 deg (equivalent to an oar blade displacement of about ±1 cm).
- Joint angle measurement sensor on the rower’s limbs: strain gauge electrogoniometers bearing the maximum dimensions up to 25 × 50 × 20 mm (L × W × H) to allow fitting on the athlete’s limbs (elbow, hip), maximum weight of 30 g, waterproof, featuring a minimum range angle of 180 deg and an accuracy of better than 1.0 deg.

In order for the sensors to take up as little space and weight as possible, a common requirement suggests using standard (5 V) power bank voltage.

### 3.3. Rowing System Fabrication and Calibration

The selection of individual sensors and the construction phases (i.e., fabrication, assembling, and calibration) of the proposed system complied with the design requirements discussed.

#### 3.3.1. Monitoring Sensors Characteristics and Accessories

In the proposed approach, the monitoring system is organized in two main parts; the first part aims at logging the boat kinematics, and the second one at recording the athlete’s kinetics and field parameters of the boat equipment (oars, seat).

1. Boat’s kinematic data acquisition subsystem

Reliable loggings of the boat’s kinematics require the combination of a satellite-positioning receiver and inertial sensors and their processing through real-time Kalman filtering to obtain a statistically optimal result. Today there exist numerous commercial options of blended GNSS/INS technologies of different grades. However, the key requirements for selecting such a system encompass physical limitations (i.e., low weight, portability, and water resistance), operational limitations (i.e., data source synchronization) as well as cost restrictions.

In this study we adopted a low-cost GNSS/MEMS INS unit (MTi-G-700 XSENS^®^). The system integrates a single frequency satellite-positioning receiver, three accelerometers, three gyroscopes, three magnetometers, and one barometer (Table 2). It is lightweight (60 g), water-resistant, and dust-resistant (IP67) and offers synchronization capabilities with other systems (sync in and sync out). It exports an integrated navigation solution at a sampling rate of 400 Hz.

The performance specifications of the XSENS MTi-G-700 are 1 m std in horizontal position accuracy and 0.05 m/s RMS in velocity. Furthermore, its performance characteristics for attitude determination for dynamic applications are 0.3 deg at roll/pitch and 1.0 deg at azimuth. These values refer to the benchmarking of the integrated positioning system against reference trajectories and not the internal accuracy of the system [48].

2. Athlete’s and boat’s equipment kinetic data acquisition subsystem

The performance specifications of this subsystem are particularly demanding compared to those for the boat kinematics. Specifically, certain limitations apply for the sensor housing and installation on the boat, the oars, and the athlete’s body. Moreover, considering that a number of different sensors are contributing data recordings, time synchronization becomes a key requirement. Table 3 summarizes the type, model, and performance characteristics of the sensors used in this study. They are analog, water-resistant devices powered with 5 V DC that provide output voltages (measurements) ending up in a digitizer.

The sensors included in Table 3 can be divided in two categories depending on the way their voltage output is digitized. The first category includes the sensors whose operation requires a digitizer with a port for voltage measurement from a Whinstone bridge (full/half/quarter). Contrarily, the second category includes sensors relying on a plain analog voltage output and a digitizer with an analog input port. In particular, the digitizer adopted in this study (NI 6211, National Instruments) incorporates 16 analog input channels (analog in), 2 analog output channels (analog out), 4 digital output channels (digital out), and 4 digital input channels (digital in). The NI 9237 National Instruments digitizer is a Wheatstone type bridge digitizer (full/half/quarter) with the ability to simultaneously acquire measurements on four channels. A single chassis (NI 9171) was chosen to power this digitizer via the USB interface.

#### 3.3.2. Oarlock Retrofit and Installation

**Figure 2:**
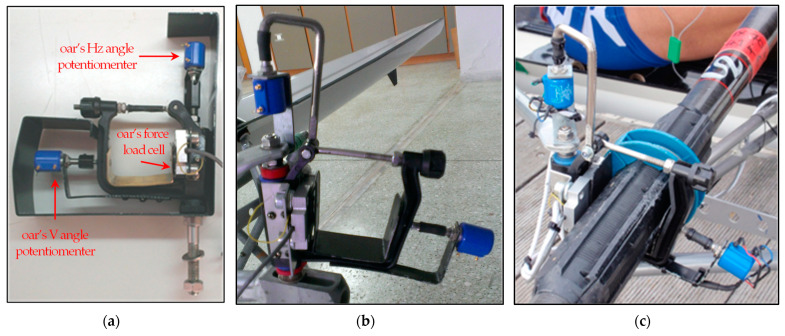
(**a**) Preliminary modified rowing oar ring, (**b**) final version of the modified rowing oar ring, and (**c**) modified rowing oar ring installed in a rowing boat.

The oar ring is a plastic assembly connected through the oar gate on the rigger extension aiming to stabilize and assist the rotation of the oar during the rowing cycle. The oar rings were suitably retrofitted to integrate two variable voltage dividers (potentiometers) for measuring the oar rotation angles (horizontal and vertical) with respect to the oar gates of the boat (Figure 2a). Furthermore, a load cell was fixed on the inner side of the oar rings to measure the applied force on the oar gate transmitted via the oar (Figure 2a). The modified oar ring was larger compared to the standard one so that could facilitate sensor placement. Oar ring modification included:

1. Oar ring retrofit: A rigid metal blade was placed on the inner side of the oar ring, where the paddle rests, to monitor its vertical movement and transfer the rotation to the potentiometer, as shown in Figure 2b.
2. Ring body machining: For the needs of retrofitting and adapting the sensors, it was prerequisite to machine the material of the ring. Figure 2a shows the first stage following ring retrofitting and machining, while Figure 2c illustrates the modified oar ring after final improvements.

#### 3.3.3. Central Unit Fabrication

The sensor controlling unit encompasses: (a) the connectors of the measuring sensors, (b) the digitizers used for converting the observed signal from analog to digital, (c) the conductors connecting the connectors to the digitizers, (d) the PC used in the role of central processing unit for the management and recording of the sensor data, and finally, (e) the battery supplying unit.

**Figure 3:**
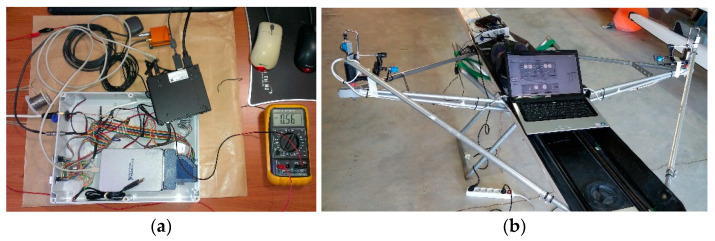
(**a**) Controller wiring check during system fabrication, (**b**) system installation test on a single scull boat, and off-water data acquisition test.

The data recording and digitization elements of the system were placed in a polycarbonate/plastic case with the dimensions 30 × 23 × 86.7 cm (L × W × H). The PC and MEMS GPS/INS subsystem operated using two lithium polymer batteries. Analog sensors were powered by a power bank so that its power supply voltage was also recorded in order to counterbalance any supply voltage drops during the stroke cycle, mainly because of the internal resistance of the voltage dividers. The connection among individual connectors and the sensors was accomplished through wiring. The wiring used to interconnect the sensors to the digitizers was carefully designed to run along the rowing boat without affecting the movement of athlete and the boat equipment (seat, oars) during the stroke cycle. At the construction stage of the monitoring system, especially during the assembling of the sensors in the control unit, particular attention was paid to ensuring continuity between (a) the sensors and the digitizers, (b) the digitizers with the micro-PC, and finally (c) the batteries with the sensors and the PC. In this way, the supply voltage of the sensors and the correct capture of records on the PC were verified (Figure 3).

At a validation stage, the functionality of the recording system was examined under real operating conditions. In this manner, the ability of the control PC to record the necessary volume of information was assessed, including the size of the raw data files. In addition, it was equally critical to control the temperature of the recording system during operational conditions, considering that the case used for housing the digital recording system was sealed to protect the electronic parts from moisture and water.

#### 3.3.4. Rowing System Setup and Calibration

Prior to on-water testing, the rowing system was tested in dry dock conditions. Off-water testing included individual controls undertaken to ensure communication and operational settings, autonomous duration capabilities, and the overall fidelity of the system according to the design specifications.

The last type of check concerned the evaluation of the fidelity of the measured quantities when transformed from the analog sensors. Specifically, a thorough checking was undertaken to verify whether the system’s recordings corresponded to the actual physical quantities (e.g., directions, displacement, and force) by converting the voltage measurements of the analog sensors. The conversion of voltage to physical quantities was achieved using the equations proposed by the manufacturer. Notably, during this check, substantial discrepancies were observed between the actual and calculated values of the measured voltage. Thus, it was considered necessary to calibrate the analog sensors.

The calibration equations recommended by the sensor manufacturers converted measured voltage (volts) to physical size (degrees, centimeters, kilograms) units derived from numerous measurements and calibration procedures using higher precision sensors. As a result, the realization of the calibration equations varied, leading to a discrepancy between the actual and calculated values of the physical quantities. For this reason, it was deemed necessary to recalibrate all analog sensors. Specifically, the following sensors were re-calibrated:

- Angle sensors placed to record instantaneous oar direction using a high precision geodetic Total Station,
- Linear displacement sensors of the seat, using a high precision geodetic Total Station,
- Force measurement sensors using a tension compression machine (MTS Insight 10 kN).

This process resulted in a number of calibration parameters for each sensor used to convert the measured voltage into natural units. In effect, discrepancies from the equations recommended by the manufacturers were observed.

### 3.4. Data Acquisition Software

#### 3.4.1. Data Logging Software

Data collection necessitates properly parameterized software, so that, depending on sensor type and principle of operation (digital or analog), it acquires and efficiently stores raw data.

The GPS/INS MEMS subsystem used for recording boat kinematics (MTi-G-700, XSENS) used MT Manager XSENS software, which allows sensor parameterization. It also allows the recording of raw data for post-processing or recording the real-time navigation solution as result of the integrated Kalman filter. The digitizers used for the Analog to Digital Conversation (ADC) were produced by the National Instruments (NI) Company. Dedicated software routines were developed in the LabVIEW environment to configure the digitizers and store recordings to the local storage unit (SSD disk).

The micro-PC integrated into the recording system offered Wi-Fi access point capabilities. Therefore, dedicated software on a portable device (tablet) was developed to assist the coach monitoring the rower’s real-time performance.

#### 3.4.2. Data Handling Software

The raw data were stored locally in the micro-PC’s disk drive in two ASCII files. At the preprocessing stage, a prerequisite is the conversion of both files into binary MATLAB^®^ files (binary .mat file). Because of the large data volume and the large number of recording sensors, this process took place in two stages. First, it included data extraction with a time stamp originating from the corresponding sensor, and second, storage of the measurements for each sensor in a separate binary .mat file.

Each one of the raw ASCII log files created at the subsystem level possessed its own structure. Thus, a script was created in Python 2.7 programming language to handle every data file. It converted logs from the GPS/INS subsystem time to UNIX time seconds and coordinates from the WGS’84 geographic coordinates to be projected in the HTRS’87 (Hellenic Transformation Reference System 1987). According to the logs that were generated from the LabVIEW data collection software that was developed, a script created two columns for each digitized channel: one with absolute UNIX time, and a second with the digitized voltage records.

**Figure 4:**
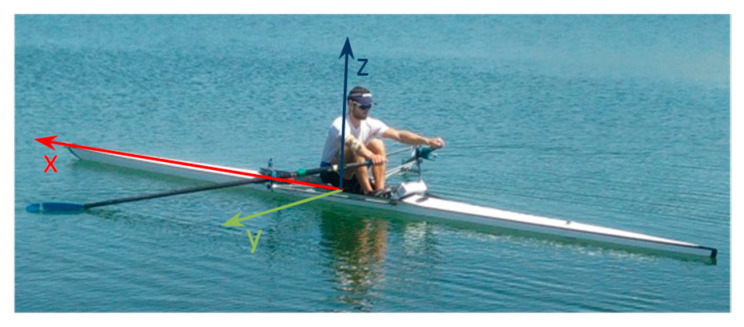
Body frame reference system projected on the rowing boat.

The last step of data preparation involved converting the boat attitude measurements (accelerations and angles) to a suitable coordinate system and data type (from ASCII to binary .mat files). The realization of the reference system (body frame) assumed its origin coinciding at the center of mass of the boat. Its parameters were defined as follows: the +*x*-axis points along the longitudinal axis of the boat in the direction of motion, the +*y*-axis points perpendicular to the *x*-axis on the right hand as the rower faces the stern of the boat, while the +*z*-axis completes the clockwise reference system—see Figure 4. In addition, course made good direction (azimuth) was defined as the clockwise angle created by the *x*-axis of the body frame from geographical north.

#### 3.4.3. Coordinate System Transformation

**Figure 5:**
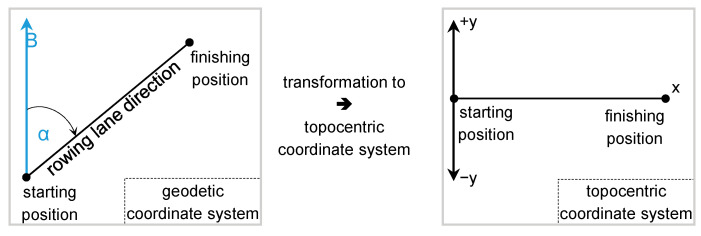
Geodetic to rowing lane topocentric coordinate system transformation.

Considering that the rowing system employed a GPS/INS receiver, the boat trajectory was originally obtained in the WGS’84 (World Geodetic System 1984) reference system, which transformed it to projected coordinates of the HTRS’87 (Hellenic Terrestrial Reference System 1987). Finally, in order to study the boat trajectory efficiently, projection coordinates were transformed to a local reference system (body frame). More specifically, this local coordinate system (Figure 5) was defined as follows: (a) its origin coincided with the initial position of the boat when placed on the rowing lane before the start, (b) the semi-axis Ox was aligned to the rowing lane, and (c) the −*yy* axis was perpendicular to the semiaxis Ox with the positive signs being on the right hand of the rower pointing the way forward of the boat.

### 3.5. Data Processing Software

#### 3.5.1. Data Logs Synchronization and Filtering

In order to analyze the rowing technique, the time series of raw data needed to be sliced and grouped into individual stroke cycles for a rowing course. The first step was the synchronization of the records as formatted from the previous stage of data handling.

Data synchronization between digital and analog sensors was originally designed using the GPS/INS pulse (Τ = 2.5 ms, f = 400 Hz) as a recording command (trigger). However, the Wheatstone bridge digitizer (NI 9237) has connectivity only for reference pulses (master timebase—f_M_) of at least 3.1 MHz to digitize with a cycle of at least 2.56 ms (390.625 Hz) according to Equation (1) [49]—and not for a trigger signal.

fs = f_M_/256/n,    n = 1, 2, …, 3 (1)

Because of this incompatibility, data synchronization between digital and analog sensors using a PC clock was mandatory. After data synchronization, data resampling took place so that the recordings of all sensors were acquired in the same time intervals between consecutive recordings for the same recording interval. Finally, for all sensors, a unique time reference was created with a fixed time interval of 2.5 ms (400 Hz) between data recordings.

Digital filtering was applied to all analog sensor recordings after synchronization. The maximum rowing rate an athlete can achieve was approximately 40 spm (strokes per min) or otherwise 40/60 strokes per second or otherwise 40/60 Hz = 0.667 Hz. Therefore, considering that periodic phenomena associated with the stroke cycle exhibited a period of less than 1 s, a cutoff frequency of 10 Hz was chosen [50]. The filter applied was a digital, low-pass Parks–McClellan filter that belongs to the FIR family [51].

#### 3.5.2. Data Logs Unit Conversion and Initialization

Analog sensor recordings were synchronized and filtered for noise; however, the measurement units remained electrical voltage (volts). Using the calibration parameters process of the analog sensors, they were converted into physical units.

Although the monitoring system is portable, it can be easily installed on different boats. After installation of the system, sensors have to be initialized in order to make the results between different boats and athletes comparable. Initialization takes place while the boat is floating at rest, and the athlete applies the predetermined position and movements of boat’s equipment, setting zero values of the analog sensors.

#### 3.5.3. Stroke Cycle Extraction and Normalization

A rowing course consists of stroke cycles, each stroke cycle being divided into phases. The execution of each phase of the stroke cycle and the transition from one phase to the next have a direct impact on the applied technique, and by extension, on the rowing performance. In order to take into account the periodicity that is inherent in the stroke cycles, it is necessary to split the entire time series of data into consecutive stroke cycles. The procedure adopted in this study assumes that the start point of every cycle coincides with the time instant the horizontal angle of the left oar is zero; that is, when the oar is perpendicular to the longitudinal axis of the boat, while still in the “recovery” phase. This assumption relies on the following observations:

1. During the “recovery” phase, the boat exhibits the highest speed during the stroke cycle and, therefore, maintains better balance.
2. The sign change of the horizontal angle (positive to negative transition) is in the midtime of the recovery phase.
3. The horizontal angle’s zero value is fixed and is independent of the athlete, the equipment settings, and the characteristics of the stroke cycle.

In order to estimate the time that the horizontal angle becomes zero, a linear interpolation between the two nearest points at sign change of the horizontal angle, is applied. After the segmentation of stroke cycles is evident, there is a need for normalizing (standardizing) the rowing cycles so that they are directly comparable. The need for standardization is critical considering the duration of the stoke cycles is not constant, either because the rower does not perform uniformly or because of the variation in the rowing rate within the stroke cycle.

**Figure 6:**
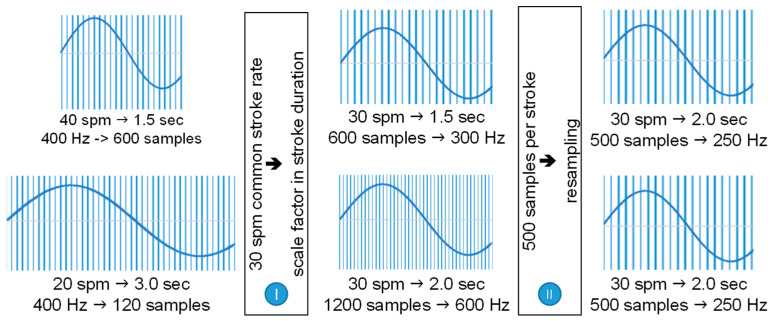
Example of normalizing two different stroke cycles (40 and 20 spm) at a reference stroke cycle of 30 spm.

Stroke cycle normalization is carried out in two stages (Figure 6) based on the reference rowing rate. In the first stage of the normalization process (I), the duration of the stroke cycle is set as equal to the duration of the reference stroke cycle. This assumption is made by imposing a deformation factor, which is calculated for each stroke cycle and applied to the duration of the stroke cycle. At the second stage of normalization (II), the timestamps are resampled in order to assign the same number of records of each observation type in each stroke cycle.

Therefore, for the case of a part of a race that includes 50 stroke cycles at an average rowing rate of 30 spm and an analog sensor recording rate of 50 kHz/31, the matrix of recordings for each measured parameter exhibits a dimension [161,290 × 1] according to Equation (2), since:

60/30 s/min/strokes/min × 50 strokes × 50,000/31 samples/s = 161,290 samples(2)

At a post normalization stage, the recording matrix counts a dimension [500 × 50] in which every column represents a stroke cycle containing 500 records. Conclusively, the normalization process allows for monitoring, comparing, and correlating consecutive rowing cycles, even for different rowers and/or races at different rowing rates.

## 4. On-Water Rowing System Operational Testing and Preliminary Results

### 4.1. Operational Testing Design of On-Water Rowing Monitoring System

After the implementation of the rowing monitoring system, a series of tests were designed to verify its completeness and operational capability in real conditions. These tests included rowing boat courses of approximately 0.5 km instead of the standard 2.0 km that has been established in rowing competitions. Courses of this length allow the athlete to repeat different rowing scenarios strictly following the coach’s instructions without becoming exhausted.

The planning of the rowing scenarios was conducted in collaboration with the coach, and their implementation carried out by an experienced rower with the ability to adapt to the coach’s instructions and suggestions. The rowing scenarios were executed on two routes (forward and backward) and were divided into the following categories.

1. Slow rowing rate—Slow and steady stroke rate (20 spm) throughout the course.
2. Rowing rate in race profile. This includes: (i) initially, 10 strokes at the fastest possible rowing rate, i.e., conditions of maximum boat acceleration at the start, (ii) 15 strokes at a fast rowing rate between 30–32 spm, i.e., constant speed conditions (maintenance), and (iii) 10 strokes with the fastest rowing rate, i.e., conditions of maximum acceleration at the finish.
3. First rowing technique error—A slow and steady rowing rate (20 spm) throughout the stroke with the athlete’s hands in the ‘recovery’ phase, returning the oars slower than normal in the ‘catch’ phase.
4. Second error in rowing technique—Slow and steady rowing rate (20 spm) throughout the stroke with the wrong technique in the ‘catch’ phase, where the athlete applies minimal force to the legs and has elevated their back.
5. Third error in rowing technique—Slow, steady rowing rate (20 spm) throughout the course with incorrect technique in the “catch” phase until just before the “recovery”, where the athlete does not apply maximum force to the oars with the required angular velocity.

Rowing data collection took place at Schinias Olympic Rowing and Canoeing Centre, Athens, Greece. The facilities of the complex were fully equipped, including a start tower, timing huts, rowing boathouses, as well as lodging facilities.

### 4.2. Rowing Data Processing

#### 4.2.1. Processing of Raw Collected Data

Data processing was exercised in two sequential steps. The first step included transforming the boat’s trajectory solution from WGS ’84 (World Geodetic System 1984) geographic coordinates to the HTRS ’87 (Hellenic Terrestrial Reference System 1987) projection coordinate system. The second step included the synchronization, filtering, initialization, and normalization processing of the stored data. In the sequel, data processing included filtering of the analog sensor recordings aiming at rejecting high-frequency noise implications. Specifically, a Parks–McClellan digital low-pass FIR filter was applied with a cutoff frequency of 10 Hz [50].

The initialization phase of the data records was particularly critical, as it converted the raw (digitized) sensor values from electrical voltage to physical quantities. The final stage concerned with data processing facilitated normalization so that the rowing cycles were standardized irrespective of the test scenario. In this regard the measured quantities were sorted by scenario and by rowing stroke cycle, so that each stroke cycle had a fixed time duration. Data normalization provided the possibility of direct comparison among different rowing stroke cycles on the same route and among different scenarios. Moreover, it facilitated computing the average rowing stroke of a course.

#### 4.2.2. Illustration of Sensors’ Processed Data

**Figure 7:**
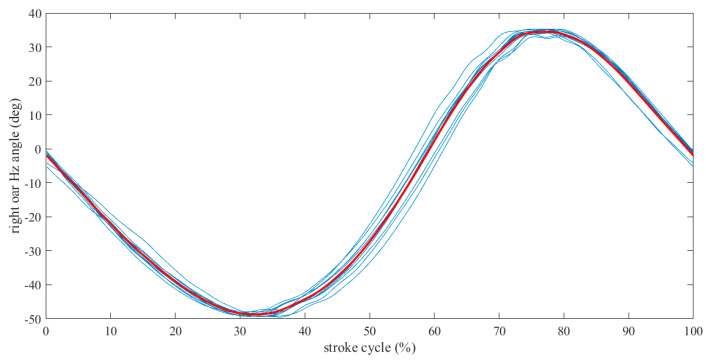
Normalized oar circles of the horizontal angle are depicted in blue, while in red is the average oar circle resulting from the average of the blue curves.

In this section, derivative plots are presented that correlate heterogeneous quantities from the combination of acquired data derived from the processed data. As an example, Figure 7 shows the horizontal angle of the right oar during the slow rowing scenario resulting from the normalization of the raw data.

**Figure 8:**
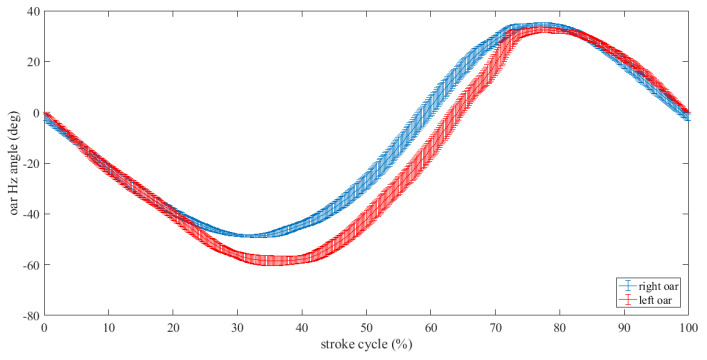
Mean stroke curves with standard deviation values superimposed of the oar horizontal angle for the low stroke rate scenario.

In particular, Figure 8 illustrates a comparison between the mean stroke oar with its standard deviation for a slow rate rowing scenario for both the left and right oars.

**Figure 9:**
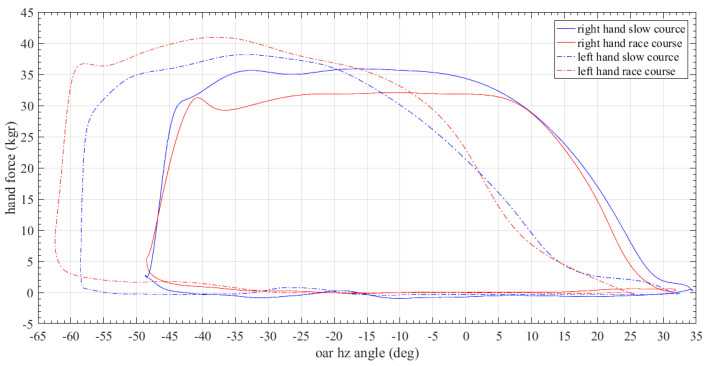
Applied hand force versus oar horizontal angle diagram.

Figure 9 shows a combined mean stroke rowing diagram representing the force applied by the athlete through the oar to the gate versus the horizontal angle of the same oar. The solid and dotted lines show the different rowing modes (solid for slow and dotted for competitive rowing rate) for the left (red) and right (blue) oars, respectively. From this plot is apparent a shift in the horizontal angle range and the profile between oars. This difference must be considered in relation to the applied force from the coach and may result in changing the oars’ settings.

**Figure 10:**
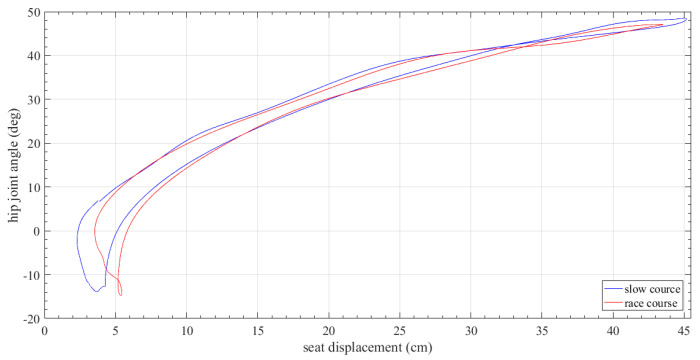
Seat displacement versus hip joint angle diagram.

Figure 10 also shows a combined mean stroke rowing diagram describing the seat position against the angle of the rower’s hip during two different rowing courses. In this case the two curves differ in the “catch” phase (hip joint angle of −15 to 10 deg) that is at the instant the athlete has bent their knees and the spoon sinks into the water. Perhaps, considering that the “catch” phase coincides with the very final stage of the stroke cycle, the athlete is bent over and cannot keep a perfect balance in the force applied in each oar.

**Figure 11:**
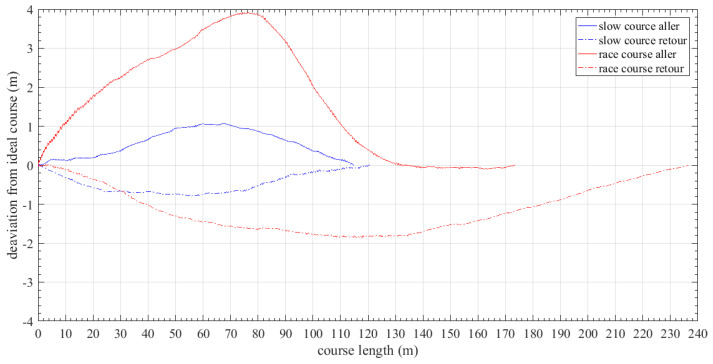
Rowing boat trajectory deviation from ideal during course.

Figure 11 shows the deviation in meters of the boat’s trajectory from the ideal path (i.e., the straight line connecting the starting point to the finish) for different courses. It is obvious that deviation was greater at racing courses since the velocity of the boat was higher, stroke cycle was shorter, and therefore the boat easily deviated from the ideal course.

**Figure 12:**
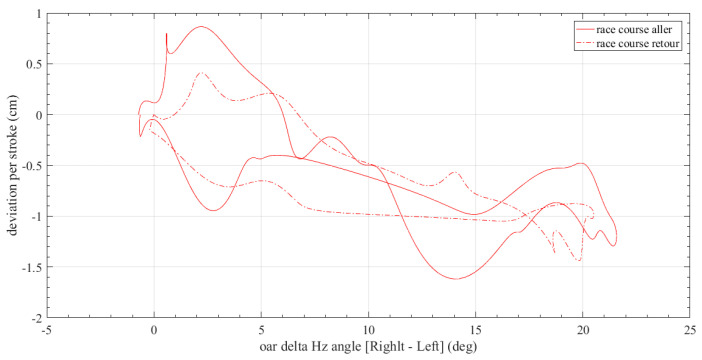
Oar’s delta horizontal angle versus rowing boat trajectory deviation.

Figure 12 depicts the differences in the oar horizontal angle (right minus left oar) against the difference computed between the actual and ideal boat direction (line joining the starting point to the end of the route) for a racing scenario. The difference at the oar’s horizontal angle is reflected in the trajectory deviation of the boat.

### 4.3. Illustration of Computed KPI and CIE Parameters

The findings in Section 4.2 demonstrated the value and the necessity of processing the data; also, many conclusions were drawn by comparing the various figures that were recorded. In the next chapter, the results of the KPIs and CIEs are visualized, as well as the result of the analysis methodology.

#### 4.3.1. KPIs Results from Rowing Testing Data

**Figure 13:**
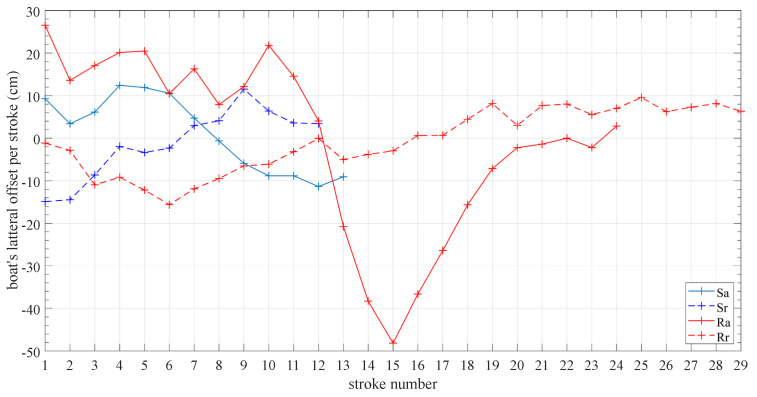
Boat’s lateral drift offset per stroke cycle.

**Figure 14:**
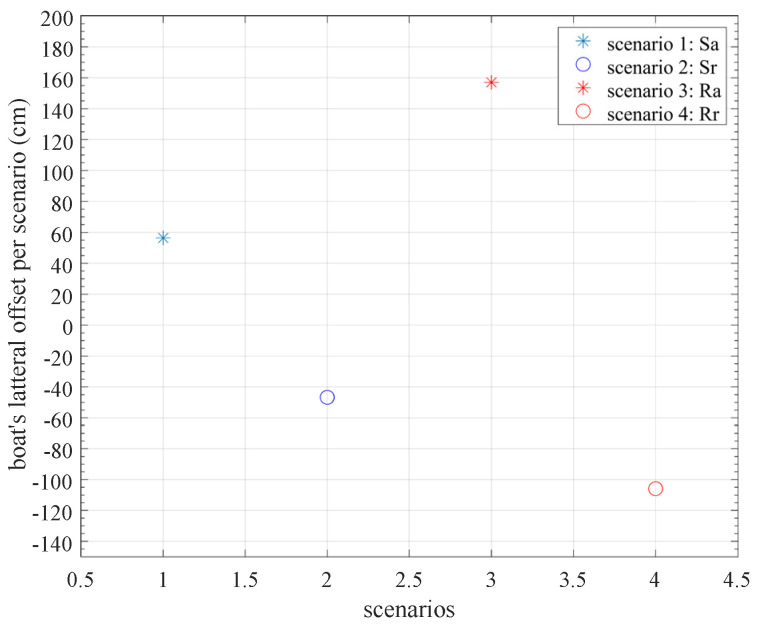
Βoat’s lateral drift offset per scenario.

Characteristic diagrams resulting from computing KPIs from the collected data were then illustrated, as described above. In the following diagrams the deflection of the boat’s position perpendicular to its direction of motion is shown per rowing cycle (Figure 13) and, on the other hand, the summarization for each rowing scenario (Figure 14).

In essence, Figure 13 and Figure 14 display the same information but in a different, more abstracted visualization in the latter. Notably, both representations are useful for the coach to accurately perceive the boat trajectory required for setting up the training instructions accordingly. For example, the error implied in the 3rd technique in the aller direction (ER3a) (Figure 14) indicates an extremely large drift offset laterally, which is consistent with the corresponding line in Figure 13. Depending on the training scenario, this information is passed to the rower in real time or at a later time to assist in improving the applied technique.

#### 4.3.2. CIEs Results from Rowing Testing Data

This section discusses the conclusions concerned with computing the athlete kinetics and the CIE parameters.

**Figure 15:**
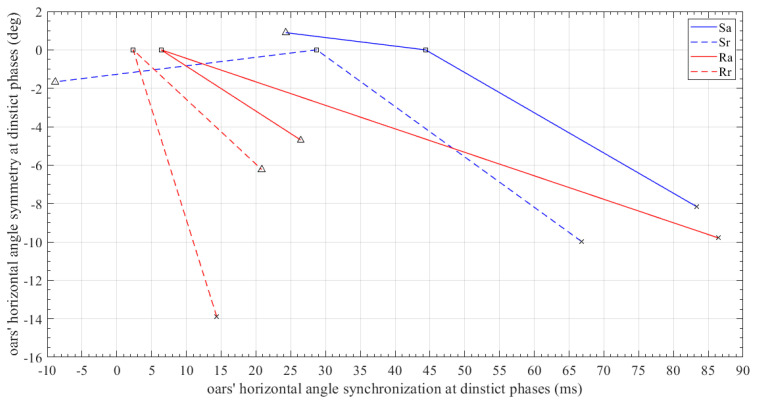
Differences in the horizontal oar angle at the entry time of the «catch», «drive», and «finish» phases versus the corresponding time lag. This diagram reveals the level of symmetry and synchronization in the horizontal oar motion.

Figure 15 illustrates the degree of symmetry achieved by the athlete in the variation of the horizontal angle of the paddle at characteristic phases of the stroke cycle—specifically, at the “catch”, “drive” and “finish” stages. In each of the three phases, the difference in the value of the horizontal angle was calculated as well as the time lag observed between the left and right oars. Each curve represents a mean rowing stroke per scenario, while the three points that define each line correspond to the three characteristic phases of the rowing cycle. “Δ”, “◻”, and “X” denote the “catch” phase, the middle of the “drive” phase, and the “finish”, respectively. Ideally, the three points of each curve should coincide at point (0, 0), as this would imply a fully symmetrical movement of the two paddles. Moreover, clearly, the smaller the area of the triangle formed by the three dots implies a higher consistency in the movement of the two oars for each stroke cycle, and thus a better coordination in the athlete performance.

## 5. Concluding Remarks and Outlook

This study introduced a new approach and an integrated recording system for performance monitoring and analysis in rowing. The basis of the proposed approach resides in the definition and computation of two sets of parameters that quantify in a holistic manner the actions that impact on the boat by the athlete (CIEs), and the responses of the boat in terms of kinematics (KPIs) characterizing the sports performance.

It was apparent from the results of the tests that the proposed monitoring system is a highly realistic approach to the rowing system. The significant innovation of the method is centered upon its ability to incorporate all different observation types into one integrated, multi-functioning sensor platform. Moreover, the system is conceptually split into several subsystems, where sensor specifications are adjusted to comply with elite rowing requirements, such as minimum space and weight, compatibility of different rowing vessels, ease of installation and robustness. Furthermore, extra care was taken to ensure high rate/fully synchronized measurements considering the high motion dynamics. Finally, by design, the system is flexible enough to expand from a single scull to a crew boat.

Preliminary testing of the system and the results obtained using extended field data verified the completeness and the correctness in terms of sensor types and setup as well as operational efficiency. Preliminary statistical analyses and data modeling undertaken based on computed CIE and KPI parameters (not presented in this paper) suggested the fruitfulness of the proposed approach and system and its potential for implementation in a broad scale.

Τhe system we developed is a prototype system that is fully functional and meets the support needs of a coach in competitive rowing. Therefore, it has the ability to record all parameters that are critical for assessing rowing performance. In the future, its expansion to record vessels with more crew and expansion in order to record secondary parameters will be deemed necessary. The development of CIE and KPI indicators was a prerequisite for the implementation of a new algorithm, briefly described in Figure 1, and concerned with the evaluation and improvement of rowers’ performances. The methodology and its results will be presented in detail in a subsequent publication.

## Figures and Tables

**Table 1:** Critical parameters for Stroke evaluation.

**kinematic**	rowing boat attitude	along/across position/velocity/acceleration body frame angles (pitch, roll, azimuth)
**kinetic**	rowing boat equipment	2D oars’ angles (horizontal, vertical), oar gate force, footstep force, seat displacement
	athlete’s body biomechanical parameters	athlete hip and elbow angles

**Table 2:** Sensors and accuracy specifications of the XSENS MTi-G-700 system (picture: [48]).

Sensor Type	Quantity	Model	Data Rate	Accuracy Specs	
GNSS receiver	1	u-blox MAX-6	4 Hz	hz accuracy: 2.5 m CEP v. accuracy: 5.0 m CEP vel. accuracy: 0.1 m/s @30 m/s	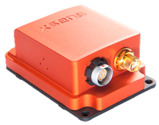
accelerometer	3	AD22293	2 kHz	bias repeatability: 0.03 m/s^2^ in-run bias stability: 40 μg noise density: 80 μg/√Hz non-orthogonal: 0.03 deg non-linearity: 0.03%FS	
gyroscope	1	ADXRS646 (for azimuth estimation)	2 kHz	noise density: 0.01°/s/√Hz non-linearity: 0.01%FS non-orthogonal: 0.05 deg	
	2	ADXRS620	2 kHz	noise density: 0.05°/s/√Hz non-linearity: 0.1%FS non-orthogonality: 0.05 deg	
magnetometer	3	na	50 Hz	noise density: 200 μG/√Hz non-linearity: 0.01%FS	
barometer	1	na	50 Hz	noise density: 0.01 hpa/√Hz	

**Table 3:** Types and specifications of sensors included in the on-water rowing monitoring system.

Category	Sub-Category	Installation Point	Type of Measurement	Digitizer	Sensor Type	Model	Performance Specifications	Photo
boat equipment	oarlock at boat gate	oarlock at boat gate	angles (Hz/V)	NI 6211	potentiometer	Bourns 3501H	linearity: ±0.25% resolution: inf	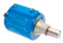
			force at oarlock	NI 9237	load cell	Omega LCPB-100	linearity: ±0.02% FS hysteresis: ±0.01% FS	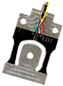
	footstep	soles in the rowing shoes	force at footstep	NI 6211	piezoelectric	Tekscan A401	linearity: <±3% FS repeatability: <±2.5% hysteresis: <3.5%FS response: <5 µs	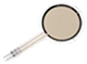
	seat	seat position	displacement		potentiometer	Celesco MT3A	accuracy: ±0.15% FS repeatability: ±0.02% FS resolution: inf	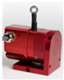
athlete body	elbow	athlete elbow	joint angle	NI 9237	strain gauge/ Wheatstone bridge	Biometrics SG110	accuracy: ±2 deg over 90 deg repeatability: <±1 deg resolution: inf	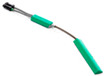
	hip	athlete hip				Biometrics SG150		

## Data Availability

Data are not available due to privacy restrictions.
